# Semirigid ureteroscopy and tamsulosin therapy as dilatation methods before flexible ureteroscopy: evaluation and benefits

**DOI:** 10.1007/s00345-023-04696-2

**Published:** 2024-02-08

**Authors:** Ahmed Issam Ali, Abdelsalam Abdelfadel, Mahmoud F. Rohiem, Ali Hassan

**Affiliations:** 1 Department of Urology, Port Said University, Port Said, 6459 Egypt; 2 Department of Urology, School of Medicine, Minia University, Minia, 61111 Egypt

**Keywords:** Flexible, Dilatation, Ureteroscopy, Tamsulosin

## Abstract

**Objective:**

To evaluate the effect of semirigid ureteroscopy and tamsulosin therapy as dilatation methods before flexible ureteroscopy advancement to the renal collecting system.

**Patients and methods:**

This prospective study included patients with renal stones less than 2 cm who underwent retrograde flexible ureteroscopy and laser lithotripsy. The patients were randomized into two groups: group A patients were given a placebo for 1 week before flexible ureteroscopy, and group B patients were administered 0.4 mg of tamsulosin once daily for 1 week before surgery and underwent active dilatation using semirigid ureteroscopy before flexible ureteroscopy. The ability of the flexible ureteroscope to reach the collecting system in both groups during the same operative session was assessed. Operative outcomes and complications were collected and analyzed in both groups.

**Results:**

A total of 170 patients were included in our study, with each group comprising 85 patients. In group B, the flexible ureteroscope successfully accessed the kidney in 61 patients, while in group A, the flexible ureteroscope was successful only in 28 cases (71.4% versus 32.9%). In group A, 33 (38.8%) patients had lower urinary tract symptoms compared to 17 (20.2%) patients in group B (*P* = 0.013).

**Conclusion:**

Using tamsulosin therapy and semirigid ureteroscopy as dilatation methods before flexible ureteroscopy increased the success of primary flexible ureteroscopy advancement to renal collecting system.

## Introduction

Through the last decade, technological advances improved endourology procedures and urinary stone management [1]. In 1912, Ureteroscopy was developed by Young, since then ureteroscopy has been used to treat urinary calculi [2]. Further technological advances allowed innovation of flexible ureteroscopes which have made retrograde intrarenal surgery an efficient and safe option to manage urinary stones [3, 4]. However, different urologists could face many obstacles while using flexible ureteroscope. One of these obstacles is failure of primary flexible ureteroscopy advancement through the ureter to the level of the renal stone [5]. The incidence of primary flexible ureteroscopy failure to reach proximal ureteral or renal stones is about 20–35% [6]. This usually leads to ureteral stent insertion for passive ureteral dilation, followed by a second ureteroscopic procedure [7, 8].

To increase the chance of FURS advancement through the ureter from the first attempt, many urologists actively dilate the ureter with balloon dilation, sequential dilators, or ureteral access sheaths before ureteroscopy. These active dilating techniques can decrease the failure rate of primary ureteroscopy to less than 5% [9]. The semirigid ureteroscope is commonly used for managing urinary stones, but there is less data regarding its use as a dilatation method to facilitate flexible ureteroscopy up to the kidney. Distal ureteral relaxation could also be achieved by alpha-one adrenergic blockers that could help in passage of FURS through the ureter [10, 11].

Ureteral mucosal damage is common during ureteral dilatation or insertion of the ureteroscope. Ureteral drainage through a double-J stent, allow these injuries to heal within 48–72 h [12].

Our study theory suggests that ureteral relaxation with alpha-1 adrenoceptor blockage and perioperative ureteral dilation with semi rigid ureteroscope could facilitate access of flexible ureteroscope up to the renal collecting system.

The purpose of this study is to evaluate the effect of using perioperative tamsulosin and semirigid ureteroscopy as dilatation methods before the advancement of flexible ureteroscopy to renal collecting system.

## Patients and methods

Our prospective randomized study included patients who presented to our outpatient clinic with renal stones suitable for treatment with FURS in the period between June 2021 and September 2022. All patients were at least 18 years old and had a renal stone burden of up to 2 cm. Pregnant women, patients with an active urinary tract infection or known ureteral stricture, multiple renal stones, and previously placed ureteral stents, and patients who had previous ureteroscopy on the same side were excluded from the study.

All included patients were randomly assigned to either group A or group B using the opaque envelope technique, with each group comprising 85 patients. Group A patients received a placebo for 1 week before flexible ureteroscopy and for another 2 weeks after the procedure. Group B patients received 0.4 mg of tamsulosin once daily for 1 week before surgery and underwent active dilatation using semirigid ureteroscopy to facilitate the advancement of flexible ureteroscopy followed by 2 weeks of oral tamsulosin intake after the procedure.

All participants were blind to the medication received and provided informed consent before surgical intervention, including counseling on treatment options, potential complications, and the need for follow-up. Preoperative data, including patient demographic and clinical data, were collected to include age, gender, body mass index, and stone characteristics. Before surgery, our patients were evaluated using non-contrast computed tomography (CT).

All surgical procedures were performed by two well-trained surgeons. Intraoperative data about each procedure were also collected, including the ability to passage flexible ureteroscopy, the amount of laser energy used through the 272-laser fiber, operative time, postoperative stenting, and intraoperative complications. All patients were followed up for 3 weeks post-operative. Any postoperative complications including and not limited to urinary tract infection, hemorrhage were classified according to the Clavien–Dindo classification. Ureteral injury if happen will be reported and classified according to Olivier Traxer classification [13].

In both groups, all procedures were performed under general anesthesia, and 2 g of cefazolin was administered during anesthesia induction. The patients were placed in the lithotomy position, and diagnostic cystoscopy was performed. A ureteric catheter size of 5 French was advanced in the ureteric orifice, and a retrograde pyelogram was performed. Next, a 0.038-in. sensor guidewire (Boston Scientific, Quincy, MA) was inserted into the kidney under fluoroscopic guidance (safety guidewire).

In group A, a double lumen ureteric catheter was inserted over the safety guidewire under fluoroscopic guidance and another 0.038- inch sensor guidewire was advanced into the kidney. A single-use flexible ureteroscope 7.4/8.4 Fr (WiScope^®^ OTU Medical Inc.) was advanced directly into the kidney over the sensor wire without active dilatation with a semirigid ureteroscope.

In group B, the semirigid ureteroscope 7 Fr, 43 cm (Karl Storz, France) was then advanced into the bladder and then into the ureter. The semirigid ureteroscope was advanced as proximally as possible, and another sensor wire was introduced through the ureteroscope up to the kidney.

After active dilation with a semirigid ureteroscope, the semirigid ureteroscope was withdrawn and a single-use flexible ureteroscope (WiScope^®^ OTU Medical Inc.) was advanced into the kidney over a sensor wire.

The entire collecting system was visualized before stone fragmentation. Stone fragmentation was done using a Holmium:YAG laser with a fiber size of 272 μm. The energy settings were 800–1200 mJ per pulse with a frequency of 8–10 Hz. The stone fragments were then removed using the Dormia basket stone extraction technique. The flexible ureteroscope was smoothly withdrawn from the ureter.

Finally, in all cases, a retrograde pyelogram was performed at the end of the procedure, and a ureteral stent (6 French, 26 cm) was placed for 2 weeks. In both groups, if the advancement of the flexible ureteroscope was unsuccessful, a ureteral stent was placed for 10–14 days before performing a second operative session.

In each case, the sum of operating time necessary to reach a stone-free status either through one or more than one session was reported.

The operative time in group A in each session was calculated from the insertion of the flexible ureteroscope till the placement of the JJ stent. The operative time in group B in each session was calculated from insertion of the semirigid ureteroscope till the placement of the JJ stent.

Then the sum of operative time in all session till reach a stone free status was reported in each patient.

The primary endpoint of the study is to report the ability of the flexible ureteroscope to reach the collecting system in both groups during the same operative session. The secondary endpoint is to determine the postoperative complications. As a result, perioperative medication was kept up for 2 weeks following surgery to assess how it affected post-operative complications. The use of the medication was unknown to all of the participants.

## Sample size

The sample size will be calculated using G* power software version 3.1.9.4 and with test family (*t* tests), type of power analysis (a priori: compute required sample size—given $\alpha$, power and effect size), input parameters, effect size = 0.64, $\alpha$ error = 0.05, power (1 − $\beta$) = 0.8 and with assuming allocation ratio *N*_1_/*N*_2_ = 1 resulting output parameters were sample size for each group is 53 patients.

### Statistical methods

Data were collected, coded, revised and entered to the Statistical Package for Social Science (Rstudio) version 2.3.2. The data were presented as number and percentages for the qualitative data, mean, standard deviations and ranges for the quantitative data with parametric distribution and median with inter quartile range (IQR) for the quantitative data with non-parametric distribution. Shapiro test was used to verify the normality of distribution.

Chi-square test was used in the comparison between two groups with qualitative data and Fisher exact test was used instead of the Chi-square test when the expected count in any cell found less than 5. Independent *t* test was used in the comparison between two groups with quantitative data and parametric distribution and Wilcoxon Mann–Whitney test was used in the comparison between two groups with quantitative data and non-parametric distribution.

The confidence interval was set to 95% and the margin of error accepted was set to 5%. So, the *P* value < 0.05 was considered significant.

## Results

**Fig. 1:**
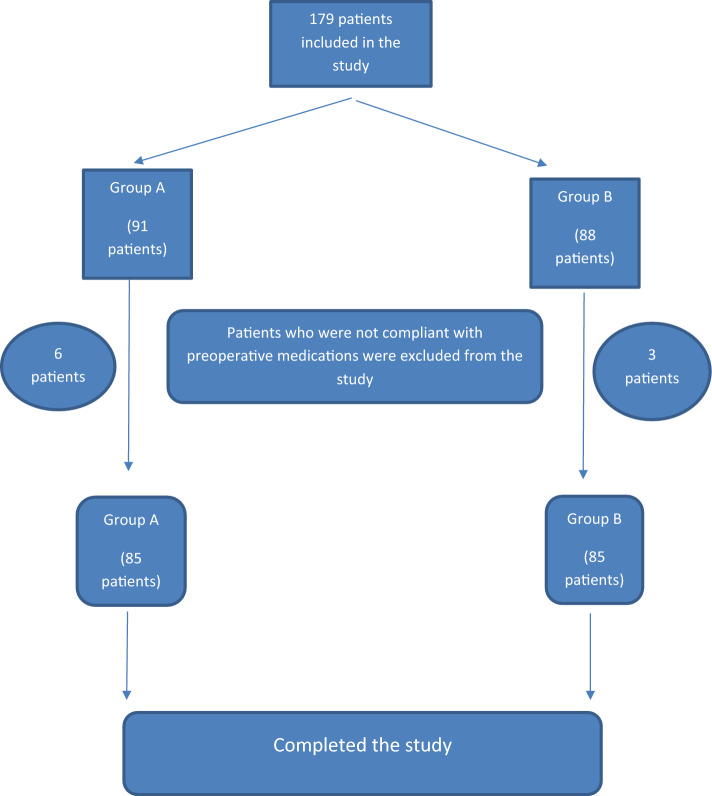
Consort diagram

A total of 170 patients who presented with renal stones and met the inclusion criteria were included in the study. The patients were randomly divided into two groups. Group A included 85 patients and group B included 85 patients (Fig. 1). There were no significant differences in patient demographics or stone characteristics between the two groups (Table 1).

**Table 1:** Pre-operative data

	Group A (*n* = 85)	Group B (*n* = 85)	*P* value
Age in years^a^			
Median (IQR)	49.0 (38.0–58.0)	49.0 (38.0–59.0)	0.739
Gender^b^			
Female	14 (16.5%)	10 (11.8%)	0.509
Male	71 (83.5%)	75 (88.2%)	
BMI^a^			
Median (IQR)	27.2 (24.5–32.2)	28.1 (24.8–32.7)	0.317
Stone Size (mm)^a^			
Median (IQR)	12.0 (10.0–14.0)	13.0 (11.0–15.0)	0.051
Stone side^b^			
Left	49 (58.3%)	52 (61.9%)	0.753
Right	35 (41.7%)	32 (38.1%)	
Stone site^c^			
Lower calyx	25 (29.4%)	26 (30.6%)	0.968
Middle calyx	15 (17.6%)	12 (14.1%)	
PUJO	2 (2.4%)	2 (2.4%)	
Renal pelvis	32 (37.6%)	34 (40.0%)	
Upper calyx	10 (11.8%)	10 (11.8%)	
Stone density^a^			
Median (IQR)	68.0 (35.0 – 88.0)	52.0 (28.0 – 86.0)	0.232
Diabetes mellitus^b^			
Yes	65 (77.4%)	61 (78.2%)	1
Hypertension			
Yes	64 (75.3%)	66 (77.6%)	0.857

^a^Mann–Whitney test

^b^Chi-square test

^c^Fisher test

In group A, the flexible ureteroscope was successful in accessing the kidney in 28 (32.9%) patients. However, in 57 (67.1) cases, the flexible ureteroscope was unsuccessful in accessing the kidney. For these patients, a JJ stent was inserted and the patients were scheduled for another session.

In group B, the semirigid ureteroscope was used as a ureteral dilator in all cases. In 61 (71.4%) cases, we were able to successfully advance the flexible ureteroscope into the kidney. However, in 24 (28.2), the flexible ureteroscope failed to pass up to the kidney, and the JJ stent was inserted and the patients were scheduled for another session (Table 2).

**Table 2:** intra operative and post-operative data

	Group A (*n* = 85)	Group B (*n* = 85)	Test of significance
Successful rate of ureteral navigation at first session			
Yes	28 (32.9%)	61 (71.8%)	Chi-square test
No	57 (67.1%)	24 (28.2%)	< 0.001*
Total operative time till a stone free status (min)			
Min.–Max.	45–105	40–84	Mann–Whitney test
Median (IQR)	66.0 (62.0–79.0)	60.0 (50.0–70.0)	< 0.001*
Laser energy			
800	60 (70.6%)	60 (70.6%)	Chi-square test
1200	25 (29.4%)	25 (29.4%)	1
Laser frequency			
8	60 (70.6%)	60 (70.6%)	Chi-square test
10	25 (29.4%)	25 (29.4%)	1
Postoperative fever			
Yes	4 (4.7%)	2 (2.4%)	Fisher test
No	81 (95.3%)	83 (97.6%)	0.678
Lower urinary tract symptoms			
Yes	33 (38.8%)	17 (20.2%)	Chi-square test
No	52 (61.2%)	67 (79.8%)	0.013*
Need for analgesia			
Yes	32 (37.6%)	21 (25.0%)	Chi-square test
No	53 (62.4%)	63 (75.0%)	0.108
Gross hematuria			
Yes	11 (12.9%)	8 (9.5%)	Chi-square test
No	74 (87.1%)	76 (90.5%)	0.646

The median operative time in group B was 60 min, which was significantly different compared to group A (66 min, *P* < 0.001). Neither group differed significantly with respect to postoperative fever, need for analgesia, gross hematuria, laser frequency, or laser energy used.

In group A, 33 (38.8%) patients had lower urinary tract symptoms compared to 17 (20.2%) patients in group B (*P* = 0.013). The complications reported in both groups were classified according to the Clavien–Dindo classification as grade 1. In both groups, ureteral injuries were limited to grade 2 as per Olivier Traxer classification, all ureteral injuries were managed successfully with JJ stent placement.

## Discussion

Flexible ureteroscopy is commonly used in treating urolithiasis due to its safety compared to percutaneous nephrolithotomy and its higher stone-free rate compared to shock wave lithotripsy [14, 15]. It is one of the first-choice treatment modalities for renal stones smaller than 2 cm in size [16]. Despite technological advances in endourology and urinary stone management in the last decade, a significant number of patients need multiple procedures due to the difficult navigation through the ureteral orifice [17, 18], which in turn decreases the success rate of Primary flexible ureteroscopy to 35% of patients with renal stones; therefore, a preoperative ureteral stent is placed for passive dilation [5, 6].

Preoperative ureteral stents improve stone-free rates and decrease operative time associated with ureteroscopy. However, there are risks associated with multiple exposures to anesthesia and prolonged ureteral stenting [19–21]. To limit the frequencies of preoperative ureteral stenting, active dilation of the ureter was required in over one-third of all endoscopic cases [6, 9], different methods of active ureteral dilatation are used including and not limited to Balloon dilation with a success rate up to 60% of cases [22, 23].

Other method of ureteral dilatation is the ureteral dilators. Mitchell et al. reported a 94% success rate of primary ureteroscopy after using ureteral dilators as an active dilatation method before primary ureteroscopy [24]. Other methods of active dilation, such as the use of ureteral access sheaths, double-lumen catheter, have been described in up to 18–26% of endoscopic cases to access the ureter [5, 6]. However, several problems are associated with active dilatation, including failure of primary ureteroscopy, high costs, possible intra operative complications [24].

Not only ureteral dilatation represents a challenge during primary ureteroscopy, but also difficult navigation through the ureteral orifice is one of the most challenging problems in primary ureteroscopy due to the narrow diameter of the orifice. One of the reasons for the narrow diameter of the ureteric orifice could be attributed to the highest concentration of alpha receptors in the distal ureteral smooth muscles which allow smooth muscle contraction. With the use of alpha-blockers, distal ureteral contraction could be inhibited and passive dilatation of the ureteric orifice is achieved [25].

Several studies have concluded that the ureteral orifice is wider in patients using alpha-blockers before ureteroscopy. This allows for shorter time needed to pass through the orifice, lower complication rates, and less mucosal damage [26–28]. Tamsulosin is the most commonly used α1 blocker in treating ureteral calculi. Sato et al. reported that tamsulosin has high affinity for α1a and α1d receptors [29]. Furthermore, tamsulosin has a rapid onset of action (4–8 h) based on *Q*_max_ after the first dose [30]. In present study, tamsulosin was given to the patients for 7 days before ureteroscopy as it achieves a therapeutic level on the 5th day [31, 32]. In present study, surgeons preferred to keep the serum blood level therapeutic for at least 3 days before the procedure to achieve maximum ureteral relaxation.

Another method of ureteral dilatation is the use of semirigid ureteroscope as a dilatation method before insertion of flexible ureteroscope to the kidney. In current study, tamsulosin therapy and semirigid ureteroscopy were used to facilitate the passage of flexible ureteroscope to the kidney, semirigid ureteroscope allows direct visualization of the ureter during ureteral dilatation.

In group B in current study, we were able to successfully navigate the ureteral orifice and advance flexible ureteroscopy into the kidney in 61 (71.8%) patients. In the other 24 patients, we failed to advance the semirigid ureteroscope into the ureter, and the ureteric stent was inserted for a second ureteroscopic session. In group A, the flexible ureteroscope was successful in accessing the kidney in 28 (32.9%) patients. However, in 57 (67.1) cases, the flexible ureteroscope was unsuccessful in accessing the kidney, and a ureteric stent was also inserted for a second ureteroscopic session.

The lower success rate in group A may be attributed to the lack of preoperative tamsulosin administration and the lack of any method of active ureteral dilatation in this group of patients with naive ureters. Different studies show lower success rate of FURS navigation to the kidney from first attempt in patient who did not receive tamsulosin or underwent any methods of active ureteral dilatation before FURS. Abdelaziz and Kidder reported in their study that, the requirement for ureteric dilatation during URS decreased after taking tamsulosin by 17.64% compared to 48.29% in the control group [28]. Another study also reported that preoperative tamsulosin decreases the requirement for ureteric dilatation from 51.6 to 32.7% [33].

In a study by Amy et al. they reported that ureteral dilatation using a semirigid ureteroscope as the only ureteral dilator before flexible ureteroscopy was successful in 98.4% of patients [34]. These results were similar to other studies of ureteral dilatation techniques before flexible ureteroscopy, which reported a 95% success rate using both balloon dilation and sequential dilators [1, 9, 22, 24]. Ureteral dilatation using a semirigid ureteroscope may offer several advantages during primary flexible ureteroscopy, many urologists may find this technique easily applicable due to the common use of semirigid ureteroscopes in routine practice. Also, using the semirigid ureteroscope allows visualization of the entire ureter, and any unexpected ureteral stones can be discovered and treated accordingly.

In addition, semirigid ureteroscopy can be passed under direct vision into the ureter, unlike balloon or sequential dilators that depend on fluoroscopic guidance for visualization. Furthermore, our technique may have some financial advantages by decreasing the high costs associated with other ureteral dilators.

The sum of operating time necessary to reach stone-free status in both groups were compared and showed that group B has an overall shorter operative time compared to group A (60 min vs 66 min, *P* value  < 0.001). The reason for the longer operative time in group A could be attributed to the inability to reach the stone from the first session in 57 (67.1%) and additional sessions were required to reach a stone free status.

In present study, there were no cases of ureteral injury or perforation in either group. In group B, there was a statistically significant reduction in the number of patients who developed lower urinary tract symptoms. Similarly another study found that taking tamsulosin during the perioperative period reduced the number of patients who experienced postoperative LUTS from 43.54 to 24.13% [33]. The same conclusion was reported by Zhu and colleagues in 2016 who found that using post-operative tamsulosin prevented LUTS in 65% of patients [35].

Ali, A. reported in his prospective study that, the incidence of hematuria in patients who undergone ureteroscopy and laser lithotripsy was decreased by utilizing tamsulosin from 22.58 to 13.79% and the incidence of postoperative fever decreased from 6.45 to 3.44% in the tamsulosin group. [33]. In our study, the use of tamsulosin decreased the incidence of hematuria from 12.9 to 9.5%. The incidence of postoperative fever decreased from 4.7% in patients receiving placebo to 2.4% in the tamsulosin group. However, the results for fever and hematuria were not statistically significant.

The present study has some limitations. First, the surgeons were not blinded throughout the study, and subsequently the surgical procedures' outcomes may be affected. Second, postoperative stent-related symptoms were not assessed using a validated questionnaire. Furthermore, our study was conducted in a single center with possible selection bias, accordingly, our study findings should be validated through a multi-institutional prospective randomized trial to limit possible study bias.

## Conclusion

Tamsulosin therapy and semirigid ureteroscopy are effective and safe methods of ureteral dilatation before flexible ureteroscopy and are associated with deceased operative times and a higher success rate of flexible ureteroscopy navigation to the kidney at the first surgical attempt.

## Data Availability

Data availability will be through contacting the corresponding author, Ahmed I Ali. ahmedissam1979@gmail.com, 00966561913009.

## Declarations

### Human and animal participants

All procedures performed in the study involving human participants were in accordance with the ethical standards of the institutional research committee and with the 1964 Helsiniki Declaration and its later amendments. The study was approved by the Bioethical Committee of Port Saied University (ERN:MED SO. (12) SPS/URS).
